# Supplementary material for: Do Not Leave Your Patients in the Dark—Using American College of Rheumatology and European Alliance of Associations for Rheumatology Recommendations for Vaccination in Polish Adult Patients with Autoimmune Inflammatory Rheumatic Diseases
Source: Vaccines (Basel). 2023 Dec 14;11(12):1854. doi: 10.3390/vaccines11121854 (PMC10748174; doi:10.3390/vaccines11121854)
Supplement: Supplementary file 1 [file vaccines-11-01854-s001.zip › vaccines-2762815-supplementary.pdf]

Badanie stanu szczepień, postawy wobec szczepień oraz wiedzy dotyczącej szczepień u pacjentów z autoimmunologicznymi zapalnymi chorobami reumatycznymi

Prosimy wypełnić wykropkowane miejsca lub zakreślić kółkiem wybraną odpowiedź.

GRYPA

Czy według Pana/Pani wiedzy są u Pana/Pani zalecane coroczne szczepienia przeciw grypie?

TAK / NIE / NIE WIEM

Czy szczepił/a się Pan/Pani kiedykolwiek przeciw grypie? TAK / NIE

Czy szczepił/a się Pan/Pani w ciągu ostatniego roku przeciw grypie? TAK / NIE

Jeśli w ciągu ostatniego roku szczepiłeś/-łaś się przeciw grypie – jaki był powód zaszczepienia (wybierz wszystkie pasujące odpowiedzi)?

- zalecenie lekarskie

- kampania informacyjna / opinia eksperta w mediach

- opinia rodziny/znajomych

- obawa przed zachorowaniem

- chęć ochrony reszty społeczeństwa („odporność stadna")

- inne………………………

Jeśli w ciągu ostatniego roku nie szczepiłeś/-łaś się przeciw grypie – jaki był powód braku zaszczepienia (wybierz wszystkie pasujące odpowiedzi)?

- obawa o działania niepożądane

- obawa o zaostrzenie choroby reumatycznej

- obawa o nieskuteczność

- nie wiedziałem/-łam że szczepienie jest zalecane

- nie obawiam się zachorowania

- chciałem/-łam ale umówienie szczepienia było utrudnione
- chciałem/-łam ale szczepionka była odpłatna
- inne………………………

COVID-19

Czy według Pana/Pani wiedzy jest u Pana/Pani zalecane szczepienie przeciw COVID-19?

TAK / NIE / NIE WIEM

Czy według Pana/Pani wiedzy jest u Pana/Pani zalecane szczepienie przeciw COVID-19 dawką przypominającą?

TAK, DWIEMA / TAK, JEDNĄ / NIE / NIE WIEM

Czy szczepił/a się Pan/Pani przeciw COVID-19?

TAK / NIE

Czy szczepił/a się Pan/Pani przeciw COVID-19 dawką przypominającą?

TAK, DWIEMA / TAK, JEDNĄ / NIE

Jeśli szczepiłeś/-łaś się dawką przypominającą przeciw COVID-19 – jaki był powód zaszczepienia (wybierz wszystkie pasujące odpowiedzi)?

- zalecenie lekarskie

- kampania informacyjna / opinia eksperta w mediach

- opinia rodziny/znajomych

- obawa przed zachorowaniem

- chęć ochrony reszty społeczeństwa („odporność stadna")

- ograniczenia nałożone na osoby niezaszczepione

- inne………………………

Jeśli nie szczepiłeś/-łaś się dawką przypominającą przeciw COVID-19 – jaki był powód braku zaszczepienia (wybierz wszystkie pasujące odpowiedzi)?

- obawa o działania niepożądane

- obawa o zaostrzenie choroby reumatycznej

- obawa o nieskuteczność

- nie wiedziałem/-łam że szczepienie jest zalecane

- nie obawiam się zachorowania

- chciałem/-łam ale umówienie szczepienia było utrudnione

- nie chciałem/-łam być przymuszana do szczepień

- inne………………………

Czy planuje Pan/Pani szczepienie kolejną dawką szczepionki przeciw COVID-19 jeśli pojawi się takie zalecenie?

TAK / NIE

PNEUMOKOKI

Czy według Pana/Pani wiedzy jest u Pana/Pani zalecane szczepienie przeciw pneumokokom?

TAK / NIE / NIE WIEM

Czy szczepił/a się Pan/Pani kiedykolwiek przeciw pneumokokom? TAK / NIE

Jeśli szczepiłeś/-łaś się przeciw pneumokokom – jaki był powód zaszczepienia (wybierz wszystkie pasujące odpowiedzi)?

- zalecenie lekarskie

- kampania informacyjna / opinia eksperta w mediach

- opinia rodziny/znajomych

- obawa przed zachorowaniem

- chęć ochrony reszty społeczeństwa („odporność stadna")

- inne………………………

Jeśli nie szczepiłeś/-łaś się przeciw pneumokokom – jaki był powód braku zaszczepienia (wybierz wszystkie pasujące odpowiedzi)?

- obawa o działania niepożądane

- obawa o zaostrzenie choroby reumatycznej

- obawa o nieskuteczność

- nie wiedziałem/-łam że szczepienie jest zalecane

- nie obawiam się zachorowania

- chciałem/-łam ale umówienie szczepienia było utrudnione

- chciałem/-łam ale szczepionka była odpłatna

- inne………………………

TĘŻEC

Czy według Pana/Pani wiedzy jest u Pana/Pani zalecane szczepienie co 10 lat przeciw tężcowi? TAK / NIE / NIE WIEM

Czy szczepił/a się Pan/Pani w ciągu ostatnich 10 lat przeciw tężcowi? TAK / NIE

Jeśli szczepiłeś/-łaś się w ciągu ostatnich 10 lat przeciw tężcowi – jaki był powód zaszczepienia (wybierz wszystkie pasujące odpowiedzi)?

- zalecenie lekarskie
- kampania informacyjna / opinia eksperta w mediach
- opinia rodziny/znajomych
- obawa przed zachorowaniem
- inne………………………

Jeśli nie szczepiłeś/-łaś się w ciągu ostatnich 10 lat przeciw tężcowi – jaki był powód braku zaszczepienia (wybierz wszystkie pasujące odpowiedzi)?

- obawa o działania niepożądane
- obawa o zaostrzenie choroby reumatycznej
- obawa o nieskuteczność
- nie wiedziałem/-łam że szczepienie jest zalecane
- nie obawiam się zachorowania
- chciałem/-łam ale umówienie szczepienia było utrudnione
- chciałem/-łam ale szczepionka była odpłatna
- inne………………………

OPINIA DOTYCZĄCA SZCZEPIEŃ

Czy uważa Pan/Pani że szczepienia u pacjentów z chorobami reumatycznymi są skuteczne?

TAK / NIE / NIE WIEM

Czy uważa Pan/Pani że szczepienia u pacjentów z chorobami reumatycznymi są bezpieczne?

TAK / NIE / NIE WIEM

Czy uważa Pan/Pani że szczepienia mogą zaostrzyć chorobę reumatologiczną?

TAK / NIE / NIE WIEM

Czy według Pana/Pani wiedzy pacjenci reumatologiczni przyjmujący leki immunomodulujące/ leki modyfikujące przebieg choroby mogą szczepić się tzw. żywymi szczepionkami?

TAK / NIE / NIE WIEM

OTRZYMYWANA OPIEKA LEKARSKA

Czy znajduje się Pan/Pani pod opieką lekarza rodzinnego / internisty?

TAK / NIE

Jeśli znajduje się Pan/Pani pod opieką lekarza rodzinnego / internisty - czy znajduje się Pan / Pani pod stałą opieką tego samego lekarza rodzinnego / internisty?

TAK / NIE

Jeśli znajduje się Pan/Pani pod opieką lekarza rodzinnego / internisty – czy lekarz zalecił Panu / Pani szczepienie przeciwko:

- coroczne szczepienia przeciw grypie? TAK / NIE

- szczepienie przeciw COVID-19? TAK / NIE

- szczepienie przeciw COVID-19 dawką przypominającą?

TAK, DWIEMA/ TAK, JEDNĄ / NIE

- szczepienie przeciw pneumokokom? TAK / NIE

- szczepienie co 10 lat przeciw tężcowi? TAK / NIE

- unikanie szczepienia żywymi szczepionkami? TAK / NIE

Czy znajduje się Pan/Pani pod opieką reumatologiczną ambulatoryjną (w poradni/ przychodni)?

TAK / NIE

Jeśli znajduje się Pan/Pani pod opieką reumatologiczną ambulatoryjną – czy znajduje się Pan / Pani pod stałą opieką tego samego lekarza reumatologa?

TAK / NIE

Jeśli znajduje się Pan/Pani pod opieką reumatologiczną ambulatoryjną – czy lekarz zalecił Panu / Pani szczepienie przeciwko:

- coroczne szczepienia przeciw grypie? TAK / NIE

- szczepienie przeciw COVID-19? TAK / NIE

- szczepienie przeciw COVID-19 dawką przypominającą?

TAK, DWOMA/ TAK, JEDNĄ / NIE

- szczepienie przeciw pneumokokom? TAK / NIE

- szczepienie co 10 lat przeciw tężcowi? TAK / NIE

- unikanie szczepienia żywymi szczepionkami? TAK / NIE

DANE SOCJODEMOGRAFICZNE

Płeć:	Kobieta / Mężczyzna
Wiek:	………....lat
Wykształcenie	PODSTAWOWE / ŚREDNIE lub ZAWODOWE / WYŻSZE
Miejsce zamieszkania	WIEŚ / MIASTO do 100 TYS. / 100-500 TYS. / >500 TYS.
Status związku	SINGIEL / W ZWIĄZKU
Zatrudnienie	PRACUJĘ / UCZĘ SIĘ / EMERYTURA lub RENTA / NIE

Które z wymienionych określeń najlepiej opisuje sposób gospodarowania pieniędzmi w Pana/Pani gospodarstwie domowym?

1. Żyję/Żyjemy bardzo dobrze – mogę (możemy) pozwolić sobie na pewien luksus
2. Żyję/Żyjemy dobrze – starcza mi (nam) na wiele bez specjalnego oszczędzania
3. Żyję/Żyjemy średnio – starcza mi (nam) na co dzień, ale musimy oszczędzać na poważniejsze zakupy
4. Żyję/Żyjemy skromnie – muszę (musimy) na co dzień bardzo oszczędnie gospodarować
5. Żyję/Żyjemy bardzo biednie – nie starcza mi (nam) nawet na podstawowe potrzeby

Table S1 Reasons for and against getting vaccinated reported by controls.

Controls vaccinated	n=8	n=37	n=6	n=16
Reasons for getting vaccinated:	Influenza	COVID-19	Pneumococcal	Tetanus
- medical recommendations	2 (25%)	13 (35.1%)	4 (66.6%)	12 (75%)
- information campaign / expert opinion in the media	1 (12.5%)	13 (35.1%)	1 (16.7%)	1 (6.3%)
- opinion of family/friends	-	8 (21.6%)	1 (16.7%)	-
- fear of getting sick	6 (75%)	26 (70.3%)	4 (66.6%)	2 (12.5%)
- the desire to protect the rest of society	5 (62.5%)	17 (45.9%)	-	-
- mandatory vaccination	-	-	-	2 (12.5%)
- restrictions imposed on unvaccinated people	-	10 (27%)	-	-
Controls unvaccinated	n=47	n=17	n=46	n=34
Reasons against getting vaccinated:	Influenza	COVID-19	Pneumococcal	Tetanus
- concerns about side effects	21 (44.7%)	15 (88.2%)	6 (13%)	1 (2.9%)
- concern about ineffectiveness	9 (19.2%)	6 (35.3%)	-	-
- lack of knowledge that vaccination is recommended	5 (10.6%)	-	34 (73.9%)	29 (85.3%)
- no worries about getting sick	13 (27.7%)	2 (11.7%)	7 (15.2%)	5 (14.7%)
- lack of time/difficult to arrange vaccination	7 (14.9%)	-	-	-
- vaccination fee	2 (4.3%)	-	1 (2.2%)	-
- reluctance to be forced	-	2 (11.7%)	-	-
- medical contraindications to vaccination	3 (6.4%)	-	-	-
- vaccination is not recommended	1 (2.1%)	-	5 (10.9%)	-
